# How Physicians in Japan Consider Patients' Social Backgrounds in Bedside Resource Allocation Decisions

**DOI:** 10.1002/jgf2.70109

**Published:** 2026-03-11

**Authors:** Tomoari Mori, Ai Unzaki, Mizuho Suzuki, Kana Nishida, Yuko Ohnuki, Kei Takeshita

**Affiliations:** ^1^ Department of Medical Ethics Tokai University School of Medicine Isehara Kanagawa Japan

**Keywords:** accountability, bedside rationing, capability approach, distributive justice, fairness, Japan

## Abstract

**Background:**

Clinicians routinely make micro‐level allocation decisions at the bedside—how much time to spend, which tests to order, or how intensively to treat. While fairness and efficiency have been studied, little is known about how patients' social backgrounds shape these decisions under universal coverage. In Japan, where financial access and free provider choice minimize monetary barriers, bedside allocation may often occur implicitly and through local negotiation rather than explicit protocols.

**Methods:**

We conducted semi‐structured interviews with 12 physicians across internal medicine, emergency, and community care settings. Transcripts were analyzed using reflexive thematic analysis to examine how social factors—such as family support, logistics/transport, and patient capability/engagement—enter allocation reasoning.

**Results:**

Three recurring reasoning tendencies emerged: Strict Egalitarians, who minimize social factors and seek uniform plans; Contextual Pragmatists, who adjust when family or logistical support is weak; and Responsibility‐Sensitive Allocators, who weigh engagement and self‐management after addressing practical barriers. These were not fixed categories—clinicians shifted among them case‐by‐case, influenced by team norms and local capacity. Across tendencies, stewardship and balance were emphasized, yet reasoning remained largely implicit and negotiated.

**Conclusion:**

Japan's “implicit and negotiated” bedside allocation enables flexibility and trust but can obscure the ethical rationale for daily decisions. Future empirical and normative work should clarify when egalitarian, pragmatic, or responsibility‐sensitive reasoning is ethically warranted and how to make reasons transparent without impeding workflow. This study suggests the practical value of maintaining flexibility while ensuring that allocation decisions remain explainable and revisable—a stance we term “answerable flexibility.”

## 1 Background

Resource allocation in healthcare has long been examined through the lens of distributive justice and fairness. Theoretical models—most prominently Rawls's justice as fairness [1], Sen's capability approach [2], and Ruger's health capability paradigm [3]—have shaped the normative foundations of how limited goods should be shared within societies. In the medical field, these frameworks have inspired debates about the ethical justification of rationing, including bedside rationing under scarcity [4]. Yet, as Daniels and Sabin emphasized, fairness in allocation is not merely about outcomes, but about the process by which decisions are made and justified [5]. Their concept of accountability for reasonableness (A4R) demands that priority setting be transparent, relevant to reasonable stakeholders, and revisable under new evidence. While this model has influenced macro‐ and meso‐level resource governance (such as insurance coverage or hospital triage committees), it remains unclear how similar ethical reasoning operates in the micro‐contexts of bedside care.

At the bedside, physicians routinely make small but cumulative allocation choices—about how intensively to treat, which tests to order, and how much time to devote to each patient. Ubel and Goold described this phenomenon as bedside rationing, arguing that such micro‐decisions constitute a moral frontier where equity, efficiency, and patient trust intersect [4]. Although clinicians rarely frame these judgments as “rationing,” their cumulative impact shapes real access to care. Existing studies from North America and Europe reveal that physicians' reasoning varies widely: some aim to uphold strict equality, while others factor in social context, prognosis, or adherence [6]. Hurst and Slowther et al. found that “context counts” for many clinicians, who believe that fair care sometimes requires adjustment for patients facing structural or social barriers [6]. However, systematic empirical data remain scarce, particularly outside Western systems where financial incentives or explicit gatekeeping shape access more strongly.

Japan provides a distinct environment for examining these issues. Its universal health coverage, established in 1961, ensures broad access and minimal financial barriers at the point of care [7]. Patients may freely choose their providers without general practitioner (GP) gatekeeping—a form of free access unparalleled among OECD countries [8]. Consequently, healthcare use is high: approximately 11 physician visits per capita annually, nearly twice the OECD average, and longer hospital stays (16 vs. 7.7 days) [8].

While such accessibility promotes equity, it also places pressure on limited human and material resources. In Japan—where universal health coverage allows free access to providers without general practitioner gatekeeping and where patient cost‐sharing is comparatively limited—bedside allocation is often addressed through team‐based coordination and locally situated clinical judgment rather than through formalized triage rules, shaped by staffing conditions and patient–clinician relationships [7, 8, 9].

Despite extensive research on the social determinants of health—notably by Marmot and colleagues [10]—there is limited empirical evidence on how clinicians translate social information into concrete bedside allocations of time, tests, and treatment intensity under universal coverage. Empirical accounts rarely track within‐clinician, case‐by‐case variation in bedside reasoning. Describing these features is important for understanding bedside decision‐making in systems where equity is structurally assumed yet practically negotiated.

Accordingly, this study examines how Japanese physicians consider patients' social backgrounds—such as social support, logistics, and self‐management capability—in everyday bedside allocation. By descriptively mapping recurring tendencies and the contexts in which they arise, we aim to provide a neutral empirical basis for subsequent dialogue on fairness, accountability, and the responsible use of flexibility in daily clinical care.

## 2 Methods

### 2.1 Study Design and Setting

We conducted a qualitative interview study to examine how physicians in Japan incorporate patients' social backgrounds into bedside resource allocation. Interviews were carried out between December 2024 and March 2025 at university hospitals, community hospitals, and private clinics in Japan. Japan's universal coverage context is described in the Background.

### 2.2 Participants and Recruitment

Participants were physicians engaged in direct patient care across multiple specialties. We used purposive sampling to capture diversity in specialty, career stage, and institutional context (university hospitals, community hospitals, and private clinics), complemented by snowball sampling and acquaintance‐based referral. Eligible participants were licensed physicians with ≥ 3 years of postgraduate clinical experience and regular involvement in allocation‐related decisions (e.g., test ordering, patient prioritization, discharge planning). Twelve physicians (9 male, 3 female; median clinical experience, 15 years; range, 5–35) provided written informed consent. Detailed participant characteristics are summarized in Table 1.

**TABLE 1:** Participant characteristics (P1–P12).

ID	Age range	Sex	Specialty/practice area
P1	50–54	F	Neurology; Genetic Medicine
P2	35–39	F	Emergency; Home Care; Occupational & Public Health
P3	65–69	M	Nephrology; General Medicine
P4	60–64	M	Intensive Care Medicine
P5	55–59	M	Respiratory Medicine; General Internal Medicine
P6	55–59	M	Emergency & Critical Care
P7	35–39	M	Emergency; Cardiovascular Critical Care
P8	50–54	M	Emergency & Critical Care
P9	50–54	M	Hematology; Home Care
P10	40–44	M	Emergency & Critical Care
P11	50–54	F	Dermatology
P12	55–59	M	Emergency; General Practice

*Note:* P‐numbers map 1:1 to transcript IDs (1–12). Age ranges are 5‐year bins. “Specialty/Practice area” denotes clinical service lines (e.g., Emergency & Critical Care, Home Care) and may include multiple entries per participant; named institutions are not reported. Participants were recruited from university hospitals, community hospitals, and clinics.

### 2.3 Data Collection

Interviews were conducted in Japanese by TM in private rooms or via secure video conferencing, with written consent for participation and audio recording. Each session lasted approximately 60–90 min. The semi‐structured guide (File S1) prompted: (1) concrete episodes of allocating limited time/staff/clinical resources; (2) working meanings and criteria of ‘fairness’ (e.g., equal standards vs. context‐adjustment/prioritization); and (3) how contextual factors (family support, transportation/logistics, work conditions, health literacy/adherence) were taken up in decisions. Follow‐up probes explored reasoning, team communication/documentation, and institutional norms, without inviting normative judgments. No repeat interviews were conducted; transcripts were not returned for comment/correction. Field notes and analytic memos were written immediately after each interview. The full interview guide is provided in File S1, and detailed reporting per the COREQ checklist is available in File S3 [11].

### 2.4 Data Analysis

Audio recordings were transcribed verbatim in Japanese and anonymized prior to analysis. We followed reflexive thematic analysis (RTA) per Braun and Clarke, iterating through familiarization, initial coding, theme development, review, definition, and narrative articulation [12]. Interpretive lenses were not pre‐specified; labels used in reporting (e.g., tendencies toward context‐sensitive adjustment or responsibility‐sensitive reasoning) crystallized inductively through repeated familiarization, coding, memoing, and team‐based reflexive dialogue. These labels are descriptive reporting aids, not a priori categories, and do not presume ontological status, ethical priority, or prevalence.

TM coded the full dataset manually (spreadsheets and memos; no CAQDAS). All co‐authors engaged in iterative review and reflexive discussion to enrich and challenge interpretations. Inter‐coder reliability metrics were not calculated, as agreement coefficients are not epistemically aligned with RTA; credibility was supported by reflexive memos and an audit‐style trail summarized in File S2 [13]. We also considered negative/variant cases during refinement and judged thematic sufficiency when no substantively new patterns emerged.

After the initial thematic analysis, we sent a one‐page summary of the main themes to three purposively selected participants for factual clarification only (not for interpretive consensus). These participants were chosen because they had provided particularly clear and internally coherent accounts that exemplified distinct patterns of bedside allocation reasoning across different clinical contexts, thereby allowing us to check that our summary captured the range of accounts described in the interviews. No substantive concerns were raised, and the thematic structure remained unchanged.

Representative quotations were translated into English by bilingual researchers and checked by the first author. AI tools were not used for analytic coding, theme development, interpretive decisions, or translation; they were used only for minor English polishing during manuscript preparation, with all content reviewed by the authors. File S2 presents representative quotes with analytic notes.

### 2.5 Ethical Considerations

The protocol was approved by the Tokai University Hospital Clinical Research Review Board (Approval No. 24R118; October 22, 2024). Participation was voluntary; no personal or institutional identifiers were collected. All authors accessed only anonymized data and declared no conflicts of interest. All procedures complied with Japan's Ethical Guidelines for Medical and Health Research Involving Human Subjects and the principles of the Declaration of Helsinki.

## 3 Results

**FIGURE 1:**
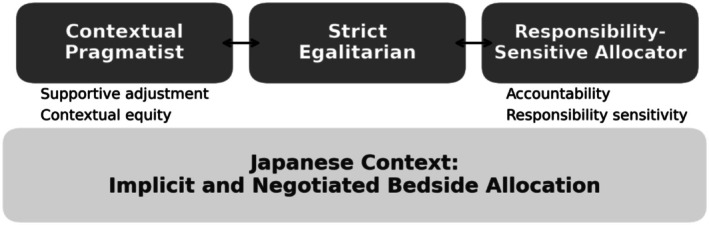
Three modes of bedside allocation reasoning in Japan.

Analysis surfaced three recurring tendencies through which clinicians allocated limited bedside resources—Strict Egalitarian, Contextual Pragmatist, and Responsibility‐Sensitive Allocator—which clinicians often moved among within a single case or across cases. Decisions were usually negotiated in relationships with patients and families and revisited in brief team dialogues. Figure 1 depicts the overall schema and its relational foundation; File S2 provides representative quotes and analytic notes.

### 3.1 Strict Egalitarian: Fairness Through Procedural Equality

Clinicians emphasized a baseline of procedural equality—“the same disease deserves the same care”—and rejected social‐status differentiation. One physician recalled: “A company executive and an older adult on public assistance were getting the same IV drip side by side—I thought, Japan is a good country.” (P5) Another affirmed that treatment content does not change by social status: “Yes, it stays the same; my concern would be capacity of the setting rather than who the patient is.” (P6) Several also noted limits: when feasibility systematically differs (living alone, transport, work constraints), identical plans can magnify unequal outcomes; in such cases, teams reconsider how to deliver “the same care” (e.g., splitting steps, adding supports, re‐sequencing explanations).

### 3.2 Contextual Pragmatist: Fairness Through Capability‐Oriented Adjustment

Participants described intentional adjustments to fit logistical and cognitive circumstances—consolidating visits or testing, simplifying regimens, and tailoring explanations. For example: “Some people simply cannot go to larger hospitals, and some struggle to understand even after repeated explanations—so we adjust accordingly.” (P11) Another noted how living situation legitimately shaped disposition decisions: “It depends on the person's circumstances… even with similar conditions, whether they can eat or move, and whether they live at home or in a facility, changes the plan.” (P7) Clinicians cautioned that unarticulated flexibility can look like favoritism; adjustments were viewed as sound when reasons were explicit and likely to improve adherence, safety, or understanding.

### 3.3 Responsibility‐Sensitive Allocator: Fairness Through Engagement and Accountability

After first addressing practical constraints (e.g., travel, costs, work schedules), clinicians calibrated the intensity and priority of follow‐up based on simple, observable indicators—continued attendance, clinically indicated self‐management, and progress toward shared goals. Two brief scenes convey the tone: “I do get irritated at nonurgent after‐hours visits, but I still say, ‘Please come in.’” (P8); and a reframing of “self‐responsibility” that maintained ordinary indicated treatment while reserving limits for costly, low‐benefit interventions (P2). Participants emphasized that any differential allocation should be minimal, explicitly reasoned, and revisable, and that structural barriers must be considered before judging effort. Some also described tension around self‐inflicted risks (e.g., persistent smoking, recreational injuries): negative emotions were acknowledged, yet clinicians prioritized clinical need in a status‐neutral manner, relying on explanation and realistic goal‐setting rather than punitive allocation (see P7 in File S2).

### 3.4 Cross‐Cutting Themes

Across modes, three ethical dynamics recurred:

1. Procedural visibility versus flexibility. Most decisions were undocumented yet often revisited in informal team dialogues; light‐touch documentation or brief team reviews were mentioned to preserve both responsiveness and accountability.
2. Capability and structural fairness. Clinicians informally assessed social support and comprehension, allocating time, explanation, and monitoring accordingly.
3. Affect and reflexivity. Negative emotions toward nonadherence or “inappropriate” use were acknowledged and tempered through reflection, helping sustain explainability and perceived fairness.

Together, these patterns converge on answerable flexibility—adaptive decision‐making that remains ethically transparent and revisable (see Figure 1; File S2).

## 4 Discussion

We identified three modes by which Japanese physicians reported incorporating patients' social background into bedside resource allocation: strict egalitarian, contextual pragmatist, and responsibility‐sensitive allocator [1, 2, 14]. We did not assign clinicians to single types, as the modes were case‐dependent rather than person‐dependent. In participants' accounts, the mode of reasoning varied by case, and the same clinician could describe different modes across different situations; however, clear criteria or triggers for such shifts were not identified in this study [4]. Illustrative quotes are provided in File S2, and the modes correspond to Figure 1.

The strict egalitarian mode emphasized impartiality and the exclusion of social information, aligning with a Rawlsian notion of formal equality under comparable circumstances [1]. The contextual pragmatist mode resonated with the capability approach: when capability or access was constrained, clinicians adjusted plans to secure real opportunities to achieve clinical goals, linking bedside reasoning to broader frameworks of social justice in health policy [2, 14]. The responsibility‐sensitive mode related engagement—continued attendance, feasible self‐management, and progress toward shared goals—to calibrated follow‐up and treatment intensity within practical and safety limits [15, 16, 17, 18].

Although our qualitative design cannot determine when each mode is normatively justified, prior Japanese preference studies show strong support for equal access in life‐saving and essential care and greater tolerance for individual preference or out‐of‐pocket choice in elective services [19]. Building on these findings and our data, we hypothesize, as a testable direction for future research, that clinicians may be more likely to draw on strict egalitarian reasoning in life‐saving or acute contexts, contextual pragmatism in chronic or capability‐limited care, and responsibility‐sensitive calibration when individual background or engagement is salient [19].

Fairness at the bedside was also intertwined with physicians' emotional and moral labor. Participants acknowledged not only frustration toward perceived overuse but also sympathetic concern and a sense of professional commitment in maintaining trustful relationships with patients. These affective responses were not portrayed as determining a specific reasoning mode but rather appeared to function as experiential signals prompting reflection on whether a shift in approach might be warranted. In several accounts, clinicians described shifts across modes—from a more strictly egalitarian application to contextual or responsibility‐sensitive adjustment—while describing efforts to maintain procedural integrity and everyday fairness. Such emotions appeared to serve as practical guides for reflexive reconsideration of allocation decisions rather than as direct determinants of a particular mode.

At the institutional level, systems such as the UK's health technology assessment framework and Korea's long‐term care system use explicit, criteria‐based entry and transparent (but proportionate) processes [20, 21]. By contrast, Japan's universal coverage and free access minimize financial barriers yet generate high utilization and persistent micro‐allocation dilemmas [7, 8]. Under these conditions, physicians often rely on informal bedside negotiation with patients and nurses. Such negotiation can be read as a culturally embedded form of relational autonomy—decision‐making shaped through mutual recognition rather than isolated individual choice [22]. It can support flexibility, trust, and workflow fit, but it may also lower external visibility and consistency and limit opportunities for collective learning. To complement this, light‐touch visibility (minimal, non‐disruptive ways of making reasons findable) may be helpful, a theme we take up in the A4R section [20, 21, 22].

From the perspective of Accountability for Reasonableness (A4R), the task is not to formalize every decision but to keep routine reasoning transparent, relevant, and revisable in proportion to workload [5, 23]. To know when each mode is ethically warranted—or not—we first need to describe, observe, and examine what actually happens at the bedside. In this spirit, a one‐sentence note in the chart or a brief peer check‐in can provide light‐touch visibility that enables appraisal without adding new rules [24, 25, 26]. This stance aligns with clinical stewardship and high‐value, cost‐conscious care by supporting the minimal, non‐disruptive record that still permits later review [9, 27]. We describe this as a stance we term “answerable flexibility”—keeping reasons lightly visible so that contextual judgment remains possible while accountability is preserved.

## 5 Limitations

This qualitative study involved twelve physicians recruited through purposive and convenience sampling within specific institutional contexts in Japan and did not include patients' or nurses' perspectives. The sample was predominantly male, largely composed of physicians over 50 years of age, and included substantial representation from emergency and critical care specialties. These characteristics may have shaped how bedside allocation reasoning and associated emotional responses were articulated, as clinicians in high‐acuity settings may encounter resource constraints more frequently and senior physicians may provide more reflective accounts grounded in accumulated clinical responsibility and practical decision‐making experience.

Our participants also had heterogeneous career trajectories spanning multiple regions, institutional settings (e.g., university hospitals, community hospitals, outpatient and home‐care practice), and evolving professional roles. While these contextual backgrounds were considered as part of the interpretive context during analysis, we did not systematically stratify or compare themes by region, institutional status, or professional role. Such contextual factors may plausibly have influenced the reported reasoning patterns and should be examined explicitly in future research.

The findings are theory‐generating and intended to support transferability rather than statistical generalization; social‐desirability and recall biases are possible. We did not systematically observe real‐time clinical decisions, so some gaps may remain between reported reasoning and enacted practice.

## 6 Conclusion

Clinical micro‐allocation in Japan is not captured by a single distributive rule but combines three modes of reasoning in context‐sensitive ways. As a practical stance, answerable flexibility—a light‐touch visibility that keeps bedside reasoning transparent, briefly recorded, and open to review without undue rigidity—provides concrete material for examining what is ethically appropriate in each case while maintaining feasible, flexible, and trust‐based clinical discretion [5, 18, 20, 21, 22, 25].

## Author Contributions

T.M.: conceptualization/study design; T.M.: investigation (interviews); T.M. (lead), A.U., M.S., K.N., Y.O., K.T.: formal analysis; T.M., A.U., M.S., K.N., Y.O., K.T. (iterative discussions, reflexive review, theme refinement): methodology/validation; T.M.: w riting – original draft; A.U., M.S., K.N., Y.O., K.T.: writing – review and editing; T.M.: supervision; All authors reviewed and approved the final manuscript.

## Funding

This work was supported by internal departmental resources; no external funding was received.

## Ethics Statement

The study was approved by the Tokai University Hospital Clinical Research Review Board (Approval No. 24R118; October 22, 2024). Participation was voluntary, and all interviewees provided informed consent prior to participation.

## Consent

The authors have nothing to report.

## Conflicts of Interest

The authors declare no conflicts of interest.

## Supporting information


**File S1:** jgf270109‐sup‐0001‐Supplementary‐File‐S1.docx.

**File S2:** jgf270109‐sup‐0002‐Supplementary‐File‐S2.docx.

**File S3:** jgf270109‐sup‐0003‐Supplementary‐File‐S3.docx.

## Data Availability

The data that support the findings of this study are available on request from the corresponding author. The data are not publicly available due to privacy or ethical restrictions.
