# Supplementary material for: How Physicians in Japan Consider Patients' Social Backgrounds in Bedside Resource Allocation Decisions
Source: J Gen Fam Med. 2026 Mar 11;27(2):e70109. doi: 10.1002/jgf2.70109 (PMC12978944; doi:10.1002/jgf2.70109)
Supplement: Supplementary file 1 — File S1: jgf270109‐sup‐0001‐Supplementary‐File‐S1.docx. [file JGF2-27-e70109-s003.docx]

**Supplement 1. Semi-Structured Interview Guide (English Version)**

Study title: Understanding Clinicians’ Perceptions of Fairness and Its Influence on Bedside Resource Allocation in Japan

Version: 2024-09-30 (English translation for publication)

Note: The original Japanese guide is available upon request. This English version follows the structure of the approved field guide.

**A. Introduction**

• Thank the participant for their time and confirm consent to participate and record the interview.

• Explain the study purpose: to understand how clinicians perceive fairness and how such perceptions influence day-to-day resource allocation.

• Emphasize confidentiality and that there are no right or wrong answers.

**B. Background information**

• Please tell me about your current clinical role and workplace setting.

• What types of allocation-related decisions are you typically involved in (e.g., tests, admission, follow-up)?

**C. Concrete experiences of resource allocation**

• Please recall a recent case in which you had to allocate limited time, staff, or medical resources among patients.

• How did you reason through your decision in that case? What factors were most influential?

• Were there any constraints, pressures, or uncertainties that shaped your judgment?

**D. Criteria and concept of fairness**

• When you make such decisions, what does ‘fairness’ mean to you in practice?

• Do you sometimes keep equal standards for all, or do you adjust to patients’ contexts?

• How do you balance equity, feasibility, and accountability?

**E. Emotional and ethical aspects**

• Have you ever felt conflict or distress about an allocation decision?

• How did you reflect on or cope with such situations?

• What would make such decisions more ethically or emotionally supportable?

**F. Team decision-making and documentation**

• How do you share these allocation decisions with your team?

• Are there opportunities for consultation, peer review, or collective discussion?

**G. Experiences during COVID-19**

• During the COVID-19 pandemic, did you experience changes in bedside resource allocation or triage?

• What lessons or practices from that period have persisted?

• Were there any cases that remain memorable to you in terms of fairness or resource constraints?

**H. Reflection and improvement**

• In your view, what could make bedside allocation decisions more transparent or fair?

• What kind of institutional or educational support would help improve fairness in practice?

**I. Closing remarks**

• Is there anything else you would like to add or feel was not covered?

• Thank the participant again and briefly debrief about the study’s next steps.

**Probes:** Follow-up questions should explore reasoning, contextual adjustments, and perceived fairness without prompting normative answers. The interviewer may use neutral phrases such as 'Could you tell me more?' or 'How did you think about that decision?'
