# Supplementary material for: How Physicians in Japan Consider Patients' Social Backgrounds in Bedside Resource Allocation Decisions
Source: J Gen Fam Med. 2026 Mar 11;27(2):e70109. doi: 10.1002/jgf2.70109 (PMC12978944; doi:10.1002/jgf2.70109)
Supplement: Supplementary file 2 — File S2: jgf270109‐sup‐0002‐Supplementary‐File‐S2.docx. [file JGF2-27-e70109-s002.docx]

**Supplementary File S2: Representative Quotes with Analytic Notes**

Note: P-numbers map 1:1 to interview IDs P1–P12. Verbatim quotes are lightly edited for clarity while preserving meaning.

**Strict Egalitarian — Fairness through uniform procedures**

- P5: “A company executive and an older adult on public assistance were getting the same IV drip side by side—I thought, Japan is a good country.”
  Analytic note: Baseline procedural equality; rejection of status-based differentiation.
- P6: “The treatment content stays the same; I worry more about the capacity of the setting than who the patient is.”
  Analytic note: Neutrality and avoidance of arbitrariness; same indication → same plan.
- P6: “If someone lives alone or lacks transport, the same plan can’t be carried out; uniformity can become unfair.”
  Analytic note: Recognizes feasibility limits; foreseeable adherence/safety risks trigger contextual adjustment.

**Contextual Pragmatist — Fairness through capability-oriented adjustment**

- P11: “Some people cannot go to larger hospitals, and some struggle to understand even after repeated explanations—so we adjust accordingly.”
  Analytic note: Adjustments for logistics/comprehension to secure outcome fairness.
- P7: “Even with similar conditions, the plan changes by the person’s circumstances—whether they can eat or move, and whether they live at home or in a facility.”
  Analytic note: Disposition shaped by living situation/function; explicit reasons guard against favoritism.
- P2 (scene): “If tests are needed but a person can only come at night, we try to consolidate them into one visit.”
  Analytic note: Re-sequencing visits/tests/explanations; a one-sentence rationale preserves explainability.

**Responsibility-Sensitive Allocator — Fairness through engagement and accountability**

- P3 (dialysis / medical non-benefit): “When I explain there is no meaningful benefit from further dialysis, some families choose, ‘Then we don’t need to continue.’”
  Analytic note: Shared goal-setting under non-benefit; restraint is clinical, not punitive.
- P8 (after-hours, nonurgent): “I do get irritated, but I still say, ‘Please come in.’”
  Analytic note: Acknowledges affect while continuing to accept patients; maintains quality of application (explainability, perceived fairness).
- P7 (self-inflicted risk: smoking/injury): “Dependence is an illness, but smoking is like a mild suicide—making oneself worse and then asking to be rescued feels problematic.”
  Analytic note: Moral emotion toward self-chosen risk, yet status-neutral care retained; uses explanation and realistic goals rather than punitive allocation.
- P7 (status-neutral stance): “I do not differentiate the intensity of care purely by social background.”
  Analytic note: Status neutrality reaffirmed; clinical need and mitigation of constraints come first.

**Cross-cutting themes**

- Visibility vs flexibility (multiple speakers): “We rarely document these decisions, but a short rationale helps the team share why.”
- Capability and structural fairness (incl. P6): “Without family support, even a simple plan may fail.”
- Affect and reflexivity (P8, P3, P7, others): “I may feel irritated, but I check the reasons before adjusting the plan.”

| **Mode** | **Ethically sound when…** | **Ethically problematic when…** |
| --- | --- | --- |
| Strict Egalitarian | Equal feasibility with clear indications | Formal equality persists despite foreseeable adherence/safety harms |
| Contextual Pragmatist | Reasons explicit; likely to improve adherence/safety/understanding | Unarticulated flexibility appears favoritist |
| Responsibility-Sensitive | Engagement conditions effectiveness/safety; constraints addressed first; differentials minimal & revisable; indicators clear | Slides into moralism, ignores constraints, or opaque differentials |
