# Supplementary material for: How Physicians in Japan Consider Patients' Social Backgrounds in Bedside Resource Allocation Decisions
Source: J Gen Fam Med. 2026 Mar 11;27(2):e70109. doi: 10.1002/jgf2.70109 (PMC12978944; doi:10.1002/jgf2.70109)
Supplement: Supplementary file 3 — File S3: jgf270109‐sup‐0003‐Supplementary‐File‐S3.docx. [file JGF2-27-e70109-s001.docx]

**Supplementary File S3. COREQ (Consolidated Criteria for Reporting Qualitative Research) Checklist**

Note: To keep the main manuscript concise, selected information is documented in COREQ only. For items marked "Not reported in manuscript", full details are provided here in COREQ.

**1. Interviewer/facilitator**

Details: TM conducted all interviews.

Reported where in manuscript: Methods — Data Collection ("Interviews were conducted in Japanese by TM…").

**2. Credentials**

Details (COREQ-only): Methods—Research team/Author roles: TM (MD, PhD, MPH); AU (MD, PhD); MS (RN, MS); KN (MBA); YO (MD, PhD); KT (MD, PhD).

Reported where in manuscript: Not reported in manuscript.

**3. Occupation at the time of study**

Details (COREQ-only): All members of the research team were university faculty at the time of the study (disciplines spanning medicine, nursing, and health management/administration).

Reported where in manuscript: Not reported in manuscript.

**4. Gender of interviewer/facilitator**

Details (COREQ-only): TM = male.

Reported where in manuscript: Not reported in manuscript.

**5. Experience and training**

Details (COREQ-only): TM trained in qualitative interviewing and reflexive thematic analysis; prior experience with semi-structured interviews and RTA.

Reported where in manuscript: Not reported in manuscript.

**6. Relationship established with participants prior to study commencement**

Details (COREQ-only): Some participants were professional acquaintances of the interviewer; however, there were no supervisory, clinical, or treatment relationships at the time of interview.

Reported where in manuscript: Not reported in manuscript.

**7. Participant knowledge of the interviewer**

Details (COREQ-only): Participants were informed that TM is a clinician-researcher studying bedside allocation; where prior acquaintance existed, this was disclosed. Participation was voluntary and confidential.

Reported where in manuscript: Not reported in manuscript.

**8. Interviewer characteristics (bias, assumptions, interests)**

Details (COREQ-only): Reflexive stance documented via analytic memos; potential assumptions discussed in team-based reflexive dialogue.

Reported where in manuscript: Not reported in manuscript.

**9. Methodological orientation and theory**

Details: Reflexive thematic analysis (RTA) per Braun & Clarke; inductive theme development with post hoc labels.

Reported where in manuscript: Methods — Data Analysis [11].

**10. Sampling**

Details: Purposive sampling to capture diversity in specialty, career stage, and institutional context; complemented by snowball/acquaintance referral.

Reported where in manuscript: Methods — Participants and Recruitment.

**11. Method of approach**

Details: Invitations via professional networks and academic societies; snowball and acquaintance-based referral.

Reported where in manuscript: Methods — Participants and Recruitment.

**12. Sample size**

Details: Twelve physicians (9 male, 3 female; median clinical experience 15 years; range 5–35).

Reported where in manuscript: Methods — Participants and Recruitment; Table 1.

**13. Non-participation (refusals, dropouts)**

Details (COREQ-only): No refusals or dropouts occurred.
Reported where in manuscript: Not reported in manuscript.

Reported where in manuscript: Not reported in manuscript.

**14. Setting of data collection**

Details: Private rooms or secure video conferencing.

Reported where in manuscript: Methods — Data Collection.

**15. Presence of non-participants**

Details (COREQ-only): No non-participants were present during interviews.

Reported where in manuscript: Not reported in manuscript.

**16. Description of sample**

Details: Age range, sex, and specialty/practice area; named institutions not reported.

Reported where in manuscript: Methods — Participants and Recruitment; Table 1.

**17. Interview guide**

Details: Semi-structured guide prompting allocation episodes, fairness criteria, and contextual factors.

Reported where in manuscript: Methods — Data Collection; Supplementary File S1.

**18. Repeat interviews**

Details: No repeat interviews were conducted.

Reported where in manuscript: Methods — Data Collection.

**19. Audio/visual recording**

Details: Audio-recorded with participant permission.

Reported where in manuscript: Methods — Data Collection.

**20. Field notes**

Details: Field notes and analytic memos written immediately after each interview.

Reported where in manuscript: Methods — Data Collection.

**21. Duration**

Details: Approximately 60–90 minutes per interview.

Reported where in manuscript: Methods — Data Collection.

**22. Data saturation / sufficiency**

Details: Thematic sufficiency was judged when no substantively new patterns emerged.

Reported where in manuscript: Methods — Data Analysis.

**23. Transcripts returned**

Details: Transcripts were not returned for comment or correction.

Reported where in manuscript: Methods — Data Collection.

**24. Number of data coders**

Details: One primary coder (TM) with iterative team review and reflexive discussion by all co-authors.

Reported where in manuscript: Methods — Data Analysis.

**25. Description of the coding tree / process**

Details: Inductive coding with iterative theme development; see Supplementary File S2 for representative quotes, analytic notes, and audit-style trail.

Reported where in manuscript: Methods — Data Analysis; Supplementary File S2 [12].

**26. Derivation of themes**

Details: Themes were developed inductively; interpretive labels crystallized post hoc through memoing and team-based reflexive dialogue.

Reported where in manuscript: Methods — Data Analysis.

**27. Software**

Details: Manual coding using spreadsheets and memos; no dedicated CAQDAS.

Reported where in manuscript: Methods — Data Analysis.

**28. Participant checking**

Details: Post-analysis, a one-page summary of main themes was sent to three participants for factual clarification only; no interpretive consensus was sought and no substantive theme changes occurred (transcripts not returned).

Reported where in manuscript: Methods — Data Analysis.

**29. Quotations presented to illustrate the themes**

Details: Representative quotations included in Results and collated in Supplementary File S2 with participant IDs (P1–P12).

Reported where in manuscript: Results; Supplementary File S2.

**30. Data and findings consistent**

Details: Findings are supported by multiple quotations per theme and by negative/variant cases; alignment documented in S2.

Reported where in manuscript: Results; Supplementary File S2.

**31. Clarity of major themes**

Details: Three recurring tendencies (Strict Egalitarian, Contextual Pragmatist, Responsibility-Sensitive Allocator) described with sub-structure and examples.

Reported where in manuscript: Results; Figure 1.

**32. Clarity of minor themes / diverse cases**

Details: Cross-cutting dynamics (procedural visibility vs flexibility; capability/structural fairness; affect and reflexivity) and variant cases are noted.

Reported where in manuscript: Results (Cross-cutting themes); Supplementary File S2.
